# Moral Injury Beyond Burnout: A Scientometric Review of Clinician Distress and Its Blind Spots in Critical Care

**DOI:** 10.3390/healthcare14183075

**Published:** 2026-09-18

**Authors:** Helmar Bornemann-Cimenti, Istvan S. Szilagyi, Christoph Klivinyi, Kordula Lang-Illievich, Sascha Hammer

**Affiliations:** 1 Department of Anaesthesiology and Intensive Care Medicine, Medical University of Graz, 8036 Graz, Austria; helmar.bornemann@medunigraz.at (H.B.-C.);; 2 Department of Psychiatry, Psychosomatics and Psychotherapeutic Medicine, Medical University of Graz, 8036 Graz, Austria; 3 Department of Anaesthesia and Intensive Care Medicine, Klinikum Güssing, 7540 Güssing, Austria

**Keywords:** moral injury, burnout, moral distress, occupational mental health, scientometric analysis

## Abstract

Background: Moral injury is increasingly used to describe clinician distress after perceived moral transgression, forced complicity, or institutional betrayal. Its distinction from burnout and its empirical organisation in healthcare remain insufficiently defined. Methods: We combined a conceptual review with a document co-citation analysis of moral-injury literature indexed in the Web of Science Core Collection from 1961 to 2026 (20 April 2026). The network comprised 1573 cited references and 5497 co-citation links and was clustered in CiteSpace. Results: The network contained ten communities and showed strong structure (modularity Q = 0.73; mean silhouette S = 0.74). Four findings were most relevant to clinical and occupational mental health: the definitional core remains unsettled; healthcare and veteran literatures have developed as partially parallel traditions; measurement and operationalisation papers occupy disproportionately prominent positions in the citation-burst and sigma rankings; and the healthcare instrument that the network identifies as structurally prominent, the Moral Injury Symptom Scale for Healthcare Professionals, retains religious or spiritual elements that require calibration in secular and confessionally heterogeneous settings. Anaesthesiology and intensive care medicine are present in the empirical literature, but as of the search date no specialty-specific work had become an organising reference for the field; citation lag accounts for part of this. Conclusions: Moral injury should not be treated as a synonym for burnout or as a deficit of individual resilience. Future research should specify definitions, validate instruments cross-culturally, stratify by clinical role and specialty and empirically test interventions directed at the moral architecture of work.

## 1. Introduction

Two referrals reach the intensive care unit within minutes of one another, and only one bed is available. Both patients have a plausible claim on it, and both families are waiting for an answer. The decision must be made before the clinical and ethical information can be as complete as the situation deserves. What remains for the intensivist is not only the memory of risk but also the awareness of a reckoning that the system did not allow.

A critical-care nurse continues high-intensity interventions in a patient they have come to regard as beyond benefit. Each task is technically correct and is performed with the care the patient is owed, yet each also deepens the sense of participating in a plan whose underlying judgement no longer matches their own. The problem is not simply workload or compassion fatigue. It is the experience of being unable to align professional action with moral conviction.

An anaesthesiologist cancels an elective operation after hearing a productive cough on the morning of surgery. The decision is defensible in clinical terms but contested in every other respect: the patient returns to the waiting list, the surgical slot is lost, and the anaesthesiologist becomes the visible cause of an outcome no party wanted. Such episodes are familiar across high-acuity healthcare because they combine time pressure, hierarchy, scarce resources, institutional expectations and immediate consequences for patients and families.

The dominant interpretive frame for clinician distress has been burnout: emotional exhaustion, depersonalisation and reduced personal accomplishment arising from chronic occupational demands that exceed coping resources [1]. Burnout remains useful, but its remedial logic is restorative: replenish what has been depleted, reduce workload, improve recovery and strengthen organisational support [2]. Many clinicians, however, describe a different injury. They speak less of being depleted than of being made complicit, betrayed or forced to act against standards that give their work moral meaning.

Moral injury offers a vocabulary for that experience. Originating outside medicine, it was first articulated in work with military veterans as lasting harm after betrayal of what is right by a legitimate authority in high-stakes circumstances [3]. Later formulations broadened the construct to include perpetrating, failing to prevent, witnessing or learning about acts that transgress deeply held moral beliefs [4]. In healthcare, this definition has been adapted to describe the psychological, emotional and existential harm that can follow forced participation in care perceived as wrong, exposure to preventable suffering, or institutional betrayal [5].

The distinction is clinically and methodologically important. If distress is misclassified as burnout, the intervention is likely to target individual recovery. If the problem is moral injury, the necessary response includes moral repair: acknowledgement, restoration of trust, and change in the conditions that produced the injury. Psychiatry and occupational mental health are central to this debate because moral injury can overlap with depression, anxiety, post-traumatic stress, shame, guilt and suicidal thinking, while also pointing beyond individual psychopathology to the ethical infrastructure of work.

The construct remains unsettled. Cognitive, functional and bio-psycho-social-spiritual accounts coexist, and no formal consensus definition currently governs clinical healthcare research [6,7]. This creates a risk that studies measure different phenomena under the same label, or that instruments developed in one cultural and occupational setting are transferred uncritically to another. A field that is conceptually unstable but increasingly applied to healthcare needs explicit mapping of its empirical structure.

This question sits between clinical practice, ethics and health-services research. It is not enough to ask whether moral injury predicts psychiatric symptoms; the field must also ask what kind of workplace event is being named, which professional roles are exposed to it, and whether the measurement language is valid in healthcare systems with different religious, legal and organisational traditions.

This Review has two aims. First, it provides a clinician-facing clarification of moral injury and its distinction from burnout, moral distress, compassion fatigue, secondary traumatic stress and post-traumatic stress disorder. Second, it maps the intellectual structure of the moral-injury literature through document co-citation analysis, with particular attention to how healthcare entered the field and why anaesthesiology and intensive care medicine remain under-represented in the references that organise it. Document co-citation analysis is used because it answers a question that narrative synthesis cannot. A narrative review reports which works its authors consider important; a co-citation network reports which works later authors repeatedly cite together, and therefore which references actually hold a field in place. Two of the findings reported here are of that kind and could not have been obtained through narrative synthesis: the partially parallel development of the veteran and healthcare traditions and the structural dominance of measurement papers over theoretical accounts. Scoping and systematic reviews of moral injury in healthcare already exist and are themselves prominent in the network [8,9,10]; this Review does not repeat their synthesis of primary findings but adds the citational architecture within which those findings are produced and read.

## 2. Methods

The narrative component was conducted iteratively to support conceptual synthesis rather than to estimate prevalence or treatment effects. Sources were identified through targeted searches in PubMed, Web of Science and Google Scholar using combinations of moral injury, moral distress, burnout, compassion fatigue, secondary traumatic stress, post-traumatic stress disorder, anaesthesiology, intensive care, critical care and critical-care nursing. Priority was given to foundational conceptual papers, integrative or systematic reviews, healthcare-specific studies and instruments used in clinical or occupational populations. This component was not systematic: no eligibility criteria were applied, no screening was performed and no yield was reported for it. The two components are kept separate throughout the manuscript. Statements about the structure of the literature in Section 3.2, Section 3.3, Section 3.4, Section 3.5 and Section 3.6 derive from the co-citation network and are supported by it; statements resting on literature selected through narrative review and our own interpretative arguments are identified as such at the point at which they are made. References discussed in the conceptual sections were, wherever possible, selected by position in the network rather than through a narrative review. A reference was eligible if it fell in the top 25 by co-citation count, in the top 25 by burst strength, or in the top 10 by sigma, or if it had foundational monographic status established by aggregated bibliography tracking across the citing articles of the two largest clusters. Standard instruments and reference texts for the differential constructs were retained as background references because they lie outside the moral-injury network but are required for construct differentiation. For the specialty-specific subsections the scheme returned too few references for substantive discussion and hand-searching was used instead; that shortfall is itself reported as a finding in Section 3.6.

**Figure 1:**
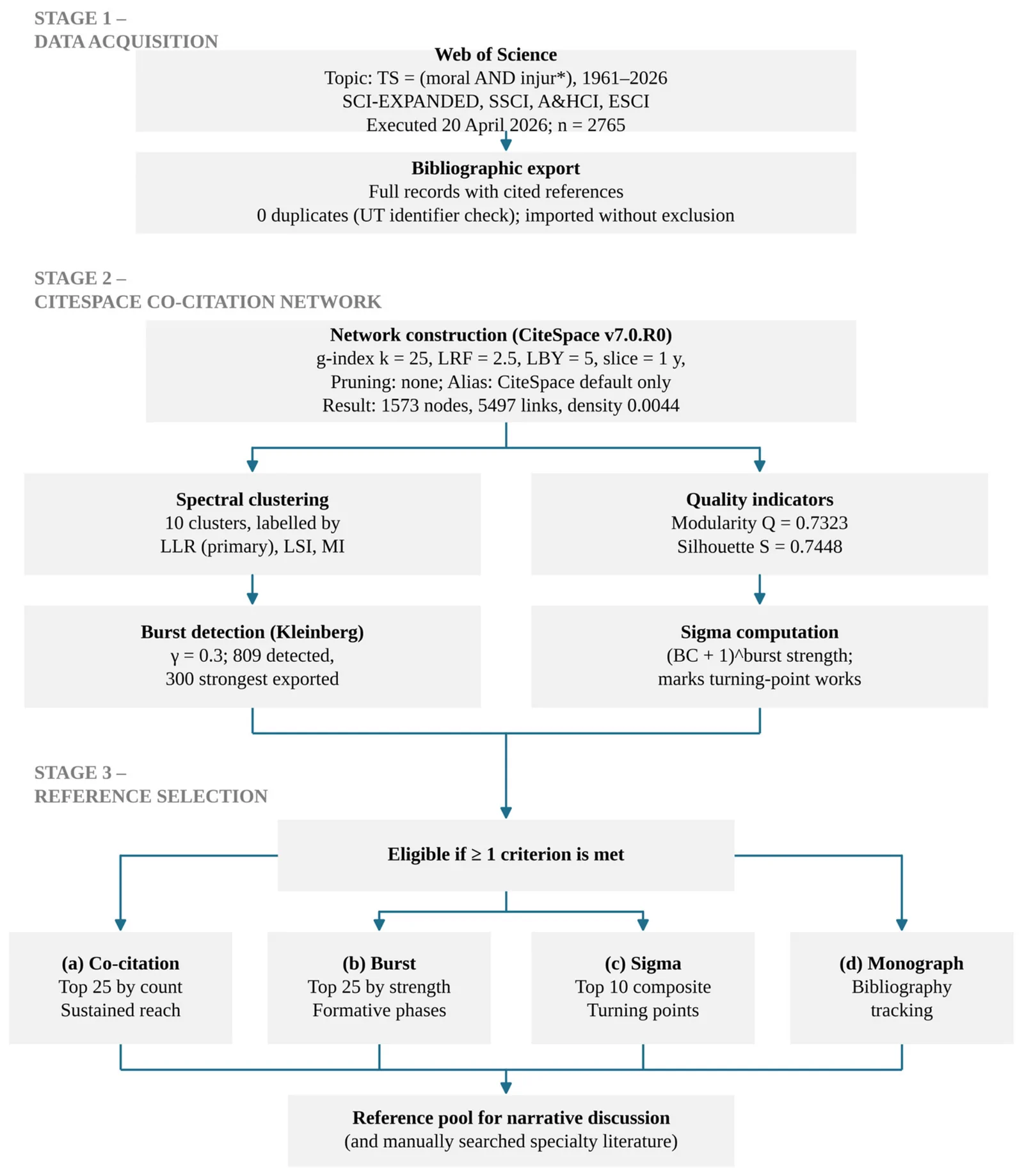
Analytical workflow of the review. Stage 1 shows record acquisition from the Web of Science Core Collection; Stage 2 shows network construction, quality assessment, burst detection and sigma computation in CiteSpace; Stage 3 shows the criteria by which references were selected for the narrative discussion.

The scientometric component used document co-citation analysis in CiteSpace version 7.0.R0 [11]. Records were retrieved from the Web of Science Core Collection using the topic query TS = (moral AND injur*), which searches title, abstract, author keywords and Keywords Plus with the Boolean AND and truncation on ‘injury/injuries/injurious’. The search covered publication years 1961–2026, spanning the SCI-EXPANDED, SSCI, A&HCI, and ESCI indexes. The search was executed on 20 April 2026 and returned 2765 source publications, all indexed as journal articles. No restrictions on language or document type were applied at export. Records were exported as full records with cited references and imported into CiteSpace without further exclusion. The 1573 nodes and 5497 links of the analysed network are cited references of these source publications, selected by the criteria below, and not the source publications themselves. The analytical workflow is summarised in Figure 1. Network nodes represented cited references and links represented pairs of references co-cited by later publications. Clusters were generated automatically and labelled by log-likelihood ratio, latent semantic indexing and mutual information. The analysis therefore examined not only how often individual references were cited but how the field linked them together over time.

Network construction used one-year time slices across the full window, with cited references as the node type and a g-index selection criterion of k = 25 within each slice; link retention was governed by a look-back window of five years and a link retaining factor of 2.5. No pruning was applied at any stage. All 2765 source publications had unique WoS accession numbers (UT identifiers), so no de-duplication of source records was required. Variant citation strings among cited references were resolved using CiteSpace’s default alias resolution only (CiteSpace version 7.0.R0 [11]), without additional manual merging. The resulting network contained 1573 nodes, 5497 links and a density of 0.0044. Clusters were extracted by spectral clustering and labelled automatically by log-likelihood ratio as the primary criterion, with latent semantic indexing and mutual information as secondary criteria.

Since the search period began in 1961, the search also identified instances of the term’s use that predate Shay’s 1994 formulation and originate from unrelated fields of legal, economic and philosophical literature. Of the 2765 source publications found, 24 (0.87%) were published before 1994; these were retained without further examination, as CiteSpace only includes a cited reference in the network if it exceeds the slice-specific g-index thresholds, and empirically speaking, only 2 of the 24 source publications from before 1994 generated network nodes with citation bursts. The network structure presented is therefore unaffected by the inclusion of these data sets. Because a Boolean conjunction across the Topic fields does not require the two terms to be adjacent, the query can also retrieve records in which ‘moral’ and an ‘injur-’ stem occur independently of the construct. We therefore classified all 2765 source records against the construct rather than assuming their relevance. Each record was assigned, under a fixed order of precedence, to one of six categories: containing ‘moral injury’ or a lexical variant as an explicit phrase; containing the two terms within three words of one another; carrying the terms non-adjacently together with at least one predefined moral-injury context marker; carrying markers of both classes; carrying no marker of either class; and carrying contamination markers only. Both marker lists are reported as regular expressions in Supplementary Table S1. Of the 2765 records, 1909 (69.04%) contained the explicit phrase, 68 (2.46%) contained the terms within three words, and 87 (3.15%) carried a context marker, so that 2064 records (74.65%) were unambiguously or contextually about moral injury. A further 23 (0.83%) carried markers of both classes, 441 (15.95%) carried no marker of either class, and 237 (8.57%) carried contamination markers only. The selection step of the analysis is protective against the remainder: a source record can influence the network only through cited references that pass the slice-specific g-index threshold, and none of the 678 records that were unmarked or carried contamination markers alone contributed a reference that passed that threshold. No node in the largest connected component, which comprises 740 references, therefore derives from them, and the cluster structure, co-citation counts, burst statistics and sigma values reported below rest on a corpus in which any contamination is confined to the periphery of the source pool.

Network quality was assessed using modularity Q and weighted mean silhouette S. Values of Q greater than 0.3 were considered meaningful and values greater than 0.7 strong; values of S greater than 0.7 were interpreted as indicating well-defined clusters. Citation bursts were detected with the Kleinberg algorithm using a sensitivity parameter of gamma = 0.3 to capture emerging bursts. Healthcare classification of bursts was performed against a predefined keyword list. Of 809 bursts detected, the 300 strongest were exported and classified. Classification was rule-based and automated rather than performed by human raters: each burst reference was matched against a predefined keyword list (nurse, clinician, hospital, healthcare, worker, COVID, pandemic, burnout, moral distress and ethics) and a predefined list of journal-abbreviation stems (NURS, MED, JAMA, BMJ, LANCET and PSYCHIATR). No inter-rater agreement statistic is therefore applicable; the classification procedure is described in the Supplementary Methods. Sigma, computed as betweenness centrality plus one raised to the power of burst strength, was used to identify turning-point references; the reference-selection scheme is set out in the Supplementary Material and summarised in Figure 1.

The 2024–2026 slices were retained in the main analysis so that the search window was reported as executed; no interpretation offered below rests on these slices, and their contribution to the reported network structure is quantified by the sensitivity analysis that follows.

To confirm the robustness of the reported network structure against citation-detection lag in the most recent slices, we re-ran the co-citation analysis with the search window restricted to publication years 1961–2023, excluding 2024–2026, for which citation accumulation is structurally incomplete. All other parameters were identical to the main analysis. The restricted analysis included 1779 source publications (down from 2765; −36%) and yielded a network of 1367 cited references and 4299 co-citation links (down from 1573 and 5497; −13% and −22%, respectively), with density 0.0046 and a largest connected component comprising 552 nodes (40%). Cluster structure was preserved: modularity was Q = 0.7648 and the weighted mean silhouette was S = 0.7645, both marginally higher than in the full network (Q = 0.7323, S = 0.7448), consistent with a sharpening of cluster boundaries once the youngest, structurally incomplete slices are removed. The dominant thematic axes were reproduced: cluster #0 was again organized around COVID-19 pandemic and healthcare-worker literature, cluster #1 around definitional, cluster #2 around the foundational operationalization work of the early 2010s, and the smaller specialty clusters (child-protection services, mechanism-focused work, mental-health training) recurred at comparable relative size. The identity and ordering of the top-ranked cited references (Griffin 2019, Bryan 2018, Williamson 2018, Farnsworth 2017, Litz 2019, Cartolovni 2021, Mantri 2020, Nash 2013 and Shay 2014) were unchanged. The findings reported below are therefore not driven by the inclusion of the most recent, structurally incomplete slices.

Interpretation proceeded in two steps. First, descriptive network features were identified from cluster size, silhouette, co-citation counts, burst strength and sigma. Second, these features were interpreted against the conceptual literature on clinician distress and occupational mental health. This separation was used to keep quantitative network description distinct from the clinical and theoretical meaning assigned to it.

The review was not designed as a prevalence review or intervention review. No pooled estimates were calculated, and no risk-of-bias assessment was applied to the conceptual literature. The purpose was to identify how the field has organised its core references and to use that structure to clarify problems of definition, operationalisation and clinical translation. The protocol was registered with the Open Science Framework (https://doi.org/10.17605/OSF.IO/NBKX9).

Because the manuscript is submitted as a Review rather than an original empirical study, the methods are reported to make the analytic logic reproducible while keeping the main emphasis on interpretation. The parameters required to reproduce the analysis are reported above rather than confined to the Supplementary Material; the CiteSpace project file and the exported network are available as stated in the Data Availability Statement.

## 3. Results

### 3.1. Moral Injury and Adjacent Constructs

Moral injury is organised around violation of moral identity and betrayal of trust. It is therefore distinct from burnout, whose dominant mechanism is a chronic imbalance between occupational demand and coping resources [1,2]. The clinician who is exhausted may require rest, workload redesign and recovery. The clinician who experiences moral injury may require acknowledgement that a wrong occurred, assurance that the conditions producing it will change, and restoration of trust in colleagues, leaders or institutions.

The contrast with moral distress is more temporal and developmental. Moral distress, introduced by Jameton and elaborated in nursing ethics, describes the situation in which a clinician knows the ethically appropriate action but is constrained from taking it [12,13,14]. Moral distress may resolve when the constraint is removed. Moral injury denotes the more persistent harm that remains after transgression or betrayal. Repeated unresolved moral distress can therefore accumulate into moral residue, the crescendo trajectory described in the nursing literature [14].

Three aetiological pathways are conventionally distinguished. Perpetration-based moral injury arises when clinicians act, or are compelled to act, against their moral standards: providing treatment they regard as futile, participating in triage decisions that leave another patient without access, or implementing a plan whose indication they contest. Witness-based moral injury follows exposure to morally troubling acts or omissions by others. Betrayal-based moral injury occurs when an authority or institution that should protect ethically defensible practice instead becomes the source of violation.

The three pathways also differ in how responsibility is experienced. Perpetration-based injury often produces self-condemnation; witness-based injury may produce anger, helplessness or disgust; betrayal-based injury often erodes trust in leaders and institutions. In healthcare these pathways can occur in the same episode. A clinician may carry formal responsibility for a decision, witness its consequences at the bedside and simultaneously experience the system as having made any ethically satisfactory alternative unavailable.

Compassion fatigue and secondary traumatic stress arise primarily from sustained empathic exposure to suffering [15,16]. Post-traumatic stress disorder is anchored in exposure to actual or threatened death, serious injury or sexual violence, with fear-based re-experiencing, avoidance and hyperarousal [17]. Moral injury can coexist with these conditions, but its dominant effects are guilt, shame, self-condemnation, loss of trust and existential disorientation. Table 1 summarises these distinctions and the intervention logic implied by each construct.

The constructs contrasted in Table 1 differ in epistemic status. Post-traumatic stress disorder is defined by codified diagnostic criteria, and burnout by an operationalisation that is established within its own literature; moral injury, moral distress and compassion fatigue are conceptual proposals whose boundaries remain contested. The entries for these three therefore represent the dominant formulations rather than agreed definitions, and the rows on temporal course, core mechanism, primary focus and intervention approach are heuristic contrasts rather than established discriminant criteria. Overlap between the constructs is the rule rather than the exception, and the table should not be read as implying settled boundaries between them.

### 3.2. Architecture of the Co-Citation Network

**Figure 2:**
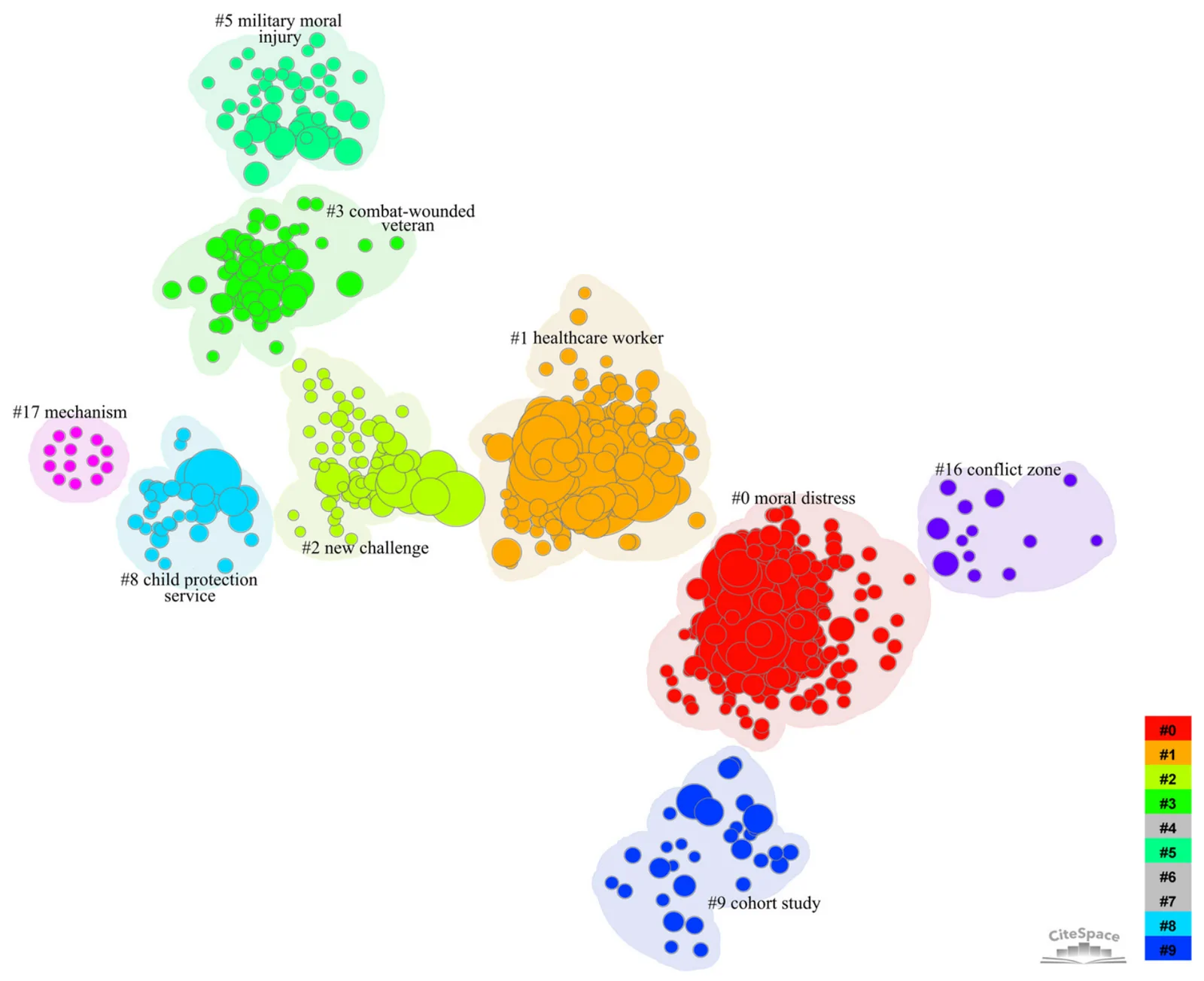
Document co-citation network of the moral-injury literature, 1961–2026. The network comprises 1573 cited references and 5497 co-citation links organised into ten major clusters; node colour indicates cluster membership, node size reflects co-citation frequency, and cluster labels are those assigned automatically by log-likelihood ratio in CiteSpace. Clusters smaller than the ten largest are omitted from the labelling for legibility.

The co-citation analysis yielded a network of 1573 cited references and 5497 links organised into ten communities. The network was strongly differentiated, with a modularity Q = 0.73 and a mean silhouette S = 0.74. Two large communities dominated the topology and together accounted for 29.5% of network nodes: clusters #0 and #1 comprised 245 and 219 nodes, respectively. The first was a healthcare and pandemic-era community anchored by healthcare moral-injury reviews, measures and commentaries [8,9,18,19,20]. The second was a veteran and definitional community built around integrative, functional and clinical accounts of moral injury [7,10,21,22,23]. The full network is shown in Figure 2.

**Figure 3:**
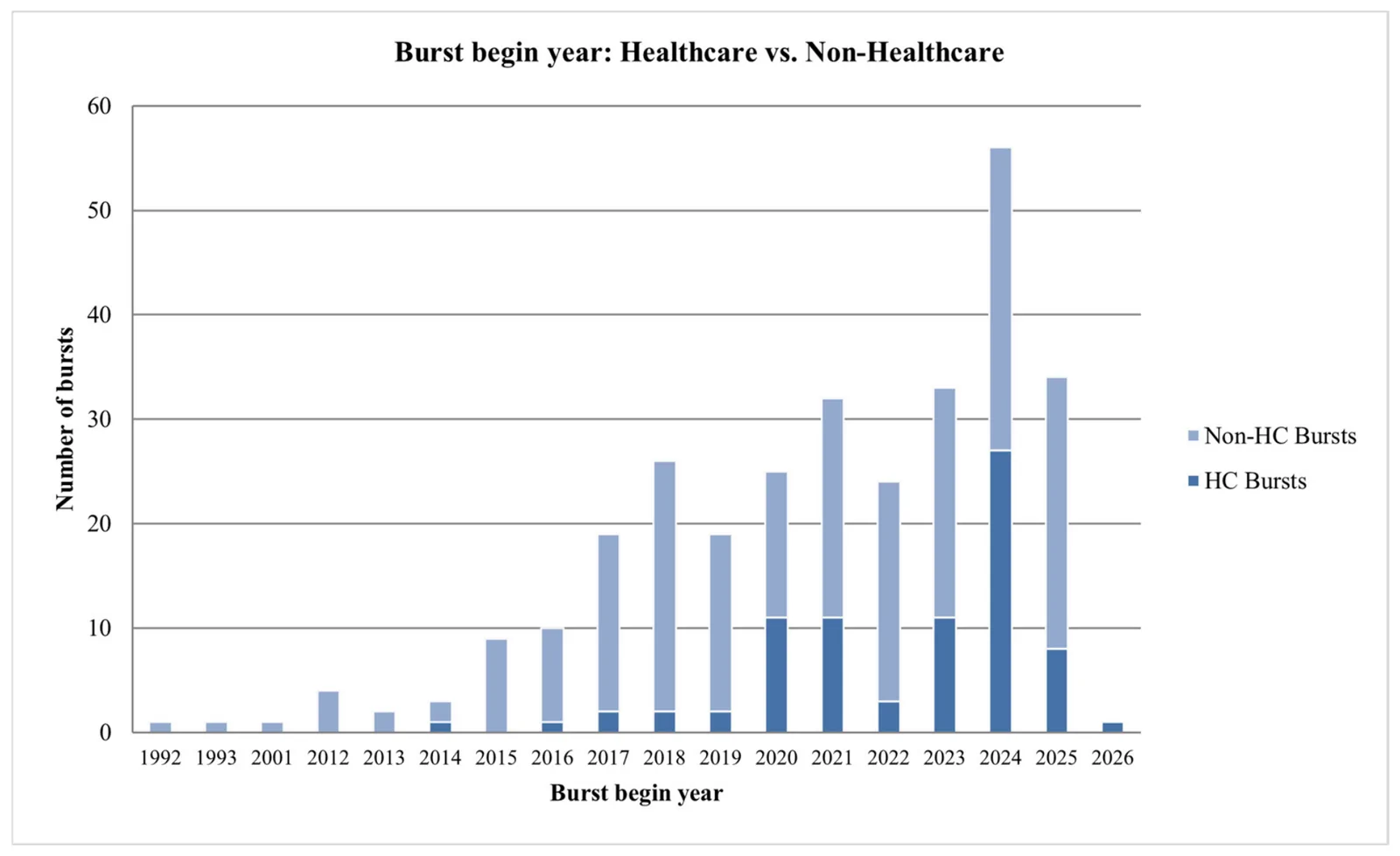
Burst-begin years in the moral-injury literature by healthcare classification. Healthcare-classified bursts are sparse before 2017, rise sharply from 2020 and reach their highest count in 2024; the 2025 and 2026 slices should be interpreted in light of citation-detection lag.

This structure suggests that healthcare moral-injury research did not simply replace the veteran literature or emerge as a later stage of the same conversation. Rather, it developed as a partially parallel formation with different entry points, populations and vocabularies. The pandemic accelerated healthcare uptake, but it did not create the construct. Healthcare-classified citation bursts were sparse before 2017 and rose sharply in 2020–2021, consistent with coronavirus disease 2019 acting as a catalyst for applying moral injury to frontline clinical work. The highest count of healthcare-classified burst onsets fell in 2024, after which citation-detection lag makes the series uninterpretable (Figure 3).

Table 2 lists the ten most co-cited references in the network. Their composition is informative. The list combines integrative reviews, healthcare scoping work, measurement papers, veteran-focused conceptual accounts and pandemic-era commentaries. It shows a field that has accumulated enough empirical and methodological density to form a recognisable network, but not enough conceptual convergence to make its definitions self-evident across settings.

The network also shows why a narrative review alone can miss structural features of a literature. A narrative account might identify the same key papers, but co-citation analysis shows how authors repeatedly link them, which clusters carry the field and where specialty literatures fail to become organising references. This is especially useful for a construct such as moral injury, where rapid clinical uptake has outpaced definitional consolidation. The ten largest clusters, with size, silhouette, mean year of cited references and automatically assigned labels, are summarised in Table 3.

### 3.3. A Field That Has Not Settled Its Terms

The definitional community had the lowest silhouette of the major clusters, indicating that the references most directly concerned with definition were co-cited with a relatively wide variety of neighbouring works. This pattern is consistent with a construct still in motion. Griffin and colleagues’ integrative review, Litz and Kerig’s special-issue framing, Hall and colleagues’ review of mental and behavioural outcomes, Bryan and colleagues’ work on post-traumatic stress disorder and suicidal behaviour, and Farnsworth and colleagues’ functional account all occupy the same definitional neighbourhood [7,10,21,22,23]. A dominant centre and an unsettled periphery are not in tension. Griffin and colleagues’ integrative review carries 269 co-citations against 148 for the next-ranked reference and functions as the field’s default point of entry; what it does not do is fix the boundaries of the construct, since the works co-cited alongside it advance cognitive, functional, betrayal-centred and bio-psycho-social-spiritual readings that are not mutually reducible. The network therefore shows convergence on what to cite together, alongside continuing divergence on what is being named.

The implication is not that these works are incompatible. Rather, they define overlapping but not identical versions of moral injury. Some foreground cognitive appraisal and self-condemnation; some emphasise betrayal and social trust; some expand the construct into bio-psycho-social-spiritual domains; and some approach it functionally as behaviour organised around moral pain [6,9,23]. Healthcare studies that do not state which account they adopt risk importing conceptual ambiguity directly into measurement and interpretation.

This matters for psychiatry because diagnostic and occupational constructs are often operationalised before their boundaries are stable. Moral injury is not currently a formal psychiatric diagnosis, and treating it as one too quickly would be premature. Yet ignoring its clinical sequelae would also be insufficient, because moral injury is associated with domains that psychiatry routinely assesses: guilt, shame, depression, anxiety, post-traumatic symptoms, loss of meaning and suicidality [9,10,22]. The field therefore requires conceptual clarity without premature diagnostic closure.

### 3.4. Two Traditions and a Shared Blind Spot

The two largest clusters also reveal a shared limitation. The healthcare cluster is organised around the generic figure of the healthcare worker or frontline clinician [8,9,18,19,20]. The veteran and definitional cluster reaches healthcare through broader occupational and trauma frameworks [7,10,21,22,23]. Both traditions therefore tend to flatten differences between specialties, professions and exposure patterns. This was understandable during the early healthcare phase, when the first task was to establish that the construct travelled beyond military settings. It becomes a limitation once the literature begins to consolidate.

Specialties differ in their moral architecture. Intensive care involves resource allocation, limitation or withdrawal of life-sustaining treatment, prognostic uncertainty and frequent family conflict. Anaesthesiology sits at the interface of elective surgery, emergency care, patient safety and hospital throughput. Psychiatry, emergency medicine, obstetrics, forensic practice and social work each carry their own configurations of moral exposure, decisional authority and institutional constraint. Aggregating these settings under the label healthcare worker produces estimates that may look precise while concealing meaningful heterogeneity.

Role differences within the same clinical setting are equally important. Critical-care nurses may have continuous bedside exposure to the consequences of decisions in which they have limited formal authority. Physicians may carry decisional responsibility but less sustained exposure to implementation. Allied health professionals may witness moral conflict while remaining peripheral to decision-making structures. These differences are not refinements; they are mechanisms. They should influence sampling, measurement, intervention design and interpretation.

The same logic applies to seniority. Trainees may experience limited agency and fear of speaking up against hierarchy, whereas consultants may experience formal responsibility for decisions constrained by institutional policy or scarcity. Leadership roles may add another layer, because leaders can be simultaneously exposed to moral conflict and perceived as the institutional source of betrayal by others. These distinctions should be visible in future datasets.

### 3.5. Instruments, Culture and Operationalisation

The strongest citation bursts were concentrated not around theoretical papers but around operationalisation. Nash and colleagues’ psychometric evaluation of the Moral Injury Events Scale carried the strongest burst, followed by Shay’s later conceptual work, Currier and colleagues’ Moral Injury Questionnaire, Bryan and colleagues’ measurement work and Drescher and colleagues’ exploratory study [24,25,26,27,28] (Supplementary Table S2). Sigma values, which combine burst strength and betweenness centrality, were likewise highest for operationalisation papers. Measurement and operationalisation papers therefore occupy disproportionately prominent positions in the citation-burst and sigma rankings. Both indicators record temporal citation attention and structural position within the network rather than intellectual influence as such, so the reading we place on the pattern, namely that instruments have shaped the field at least as much as theory has, is offered as an interpretation and not as a direct network measurement.

This finding is important because instruments carry assumptions about agency, guilt, shame, spirituality, betrayal and trauma exposure. A scale developed for military populations may emphasise transgressive acts, command structures or combat-related betrayal. A healthcare scale may emphasise professional role, patient-care obligations or religious and spiritual distress. Both may be valid within their source contexts, but neither can be assumed to measure the same construct in every clinical setting.

This pattern has practical consequences. Instruments do not merely measure a construct after it has been defined; they help determine what counts as the construct. Healthcare research that adopts military-derived or early healthcare instruments without examining their assumptions inherits this circularity. The risk is especially relevant when moral injury is studied across professions, languages and healthcare systems, because item content may reflect the cultural and institutional setting in which the instrument was created.

A related issue is the religious and spiritual signature of the operationalisations that the network identifies as structurally prominent. Foundational operationalisation clusters carry labels such as psycho-spiritual development and religious strain, and the Moral Injury Symptom Scale for Healthcare Professionals includes religious or spiritual struggles as a constitutive subscale [18]. This genealogy is coherent in United States veteran and chaplaincy-linked contexts, where religious framing of moral experience may be institutionally embedded. It may not function equivalently in secular European or confessionally heterogeneous healthcare settings. No systematic item-level analysis of the healthcare instruments in current use was undertaken. This observation therefore applies to the instruments that the network and the conceptual review identified as prominent, and it should not be generalised to healthcare instruments as a class.

The implication is not that spiritual distress is irrelevant. For some clinicians it may be central. The point is that spiritual and religious items require explicit cross-cultural calibration before prevalence, severity or interprofessional comparisons are interpreted. Research programmes that operate across languages, national healthcare systems and cultural models of distress are well placed to contribute to this work.

### 3.6. The Specialty Gap

Anaesthesiology and intensive care medicine are not absent from the empirical and commentary literature on moral injury, but they are absent from the references that organise it. None of the ten most co-cited references and none of the strongest citation-burst references originates in an anaesthesiology or intensive care journal or takes either specialty as its principal empirical site. The network is therefore connected to critical care mainly through generic healthcare and pandemic-era work rather than through specialty-specific conceptual or empirical contributions. This is a statement about the co-citation core, not about the literature as a whole. Absence from the most co-cited and strongest-burst references establishes that specialty-specific work has not become an organising reference for other authors; it does not establish that such work is missing, and it cannot establish that specialty perspectives are absent from the field’s thinking. Work of precisely this kind has appeared in leading specialty journals with moral injury as its explicit frame, in both editorial and in empirical form [29,30], without entering the co-citation core.

This gap is partly temporal. Specialty-specific work is recent, and citation-burst dynamics lag publication. It is also citational. Studies published in specialty journals may remain weakly connected to the central moral-injury literature if they do not cite or engage with the references that define the field. Closing the gap therefore requires more than adding another specialty sample. It requires linking specialty-specific moral exposures to the conceptual and measurement debates already organising the literature.

To quantify the specialty gap, we compared the empirical presence of anaesthesiology, intensive care medicine, critical-care nursing, perioperative and pain medicine (henceforth the ‘specialty group’) across three levels of the citation record (Supplementary Table S3). Classification used a single criterion at all three levels: publication in a specialty-group journal, because author affiliations are not systematically available in the cited-reference records exported by Web of Science. Of the 2765 source publications retrieved, 64 (2.31%) appeared in specialty-group journals, confirming the field’s empirical presence. Yet only seven of the 740 references in the largest connected component of the co-citation network (0.95%) were published in these journals, and none of the 300 strongest citation bursts originated in them (0.00%). Admitting a second route at the source level, namely at least one author affiliated to a specialty-group department, raises the source figure to 81 records (2.93%) and leaves the comparison unchanged. The specialty group is therefore visible in publication output, but not in the citational architecture of the field.

The citation problem matters because disconnected specialty papers can create parallel vocabularies. Anaesthesiology and intensive care medicine do not need a separate moral-injury language; they need empirical work that shows how their recurrent moral conflicts test and refine the wider construct.

For anaesthesiology and intensive care medicine, the relevant exposures include triage decisions, capacity constraints, emergency decision-making, cancellation of procedures, limitation of treatment, conflict between safety and throughput and the interprofessional mismatch between moral exposure and decisional authority. These features make the specialties productive vantage points for understanding moral injury in healthcare more broadly, not exceptional cases outside psychiatry’s concern.

The gap also has a practical consequence for intervention research. If the field continues to rely on generic healthcare categories, interventions will be designed for an average clinician who may not exist. The ethically relevant unit may be the resuscitation team, the intensive care bedside, the operating-theatre list, the emergency pathway or the governance process through which scarcity is translated into clinical decisions. Specialty-specific research can therefore strengthen, rather than narrow, the general moral-injury literature.

## 4. Discussion

This Review supports a level-specific understanding of moral injury. At the individual level, moral injury can present with guilt, shame, self-condemnation, loss of trust, existential distress and symptoms that overlap with depressive, anxiety and post-traumatic disorders. At the organisational level, it points to institutional conditions that force clinicians into morally compromised action or silence. The error is to collapse either level into the other. Treating moral injury only as personal psychopathology medicalises a structural problem; treating it only as organisational failure neglects people who may require clinical care.

The implications set out in this section are interpretative. A co-citation analysis describes how a literature is organised; it cannot show that one intervention outperforms another, and the recommendations below are therefore derived from the wider empirical and conceptual literature and from our reading of it, not from the network. This qualification applies to everything that follows in this section, including the remarks on resilience training and individual support, on psychiatric assessment, and on ethics consultation, interprofessional debriefing and organisational repair. The first implication is that resilience training cannot be the default answer. Individual support, supervision and psychotherapy may be necessary for clinicians with clinically significant symptoms. However, when the precipitating exposure is betrayal, forced complicity or ethically indefensible resource constraint, interventions that focus only on coping can intensify the injury by implying that the clinician’s reaction, rather than the situation, is the problem. The more appropriate target is the moral architecture of work: ethical infrastructure, participatory decision-making, staffing and resource conditions, as well as credible mechanisms for voicing and resolving moral concern.

For clinical services, this means that assessment should include both symptom and exposure language. Clinicians presenting with depression, anxiety, insomnia, intrusive memories or occupational withdrawal may need standard psychiatric assessment, but the history should also ask whether the distress is linked to perceived transgression, preventable suffering, silence under hierarchy or institutional betrayal. This does not replace diagnosis; it prevents the causal narrative from being lost.

Plausible organisational interventions include structured ethics consultation, interprofessional debriefing, reflective practice, Schwartz Center Rounds and leadership practices that make moral conflict visible rather than private [31]. The evidence base for moral-injury-specific interventions remains limited, and many proposed approaches have been evaluated for wellbeing, communication or organisational culture rather than for moral injury itself. The adjacent moral-distress literature points in the same direction: a scoping review with an evidence gap map found that evaluated interventions cluster on education and on ethics deliberation or consultation, are delivered predominantly at the individual or team level and act around or after the moral event, with only one of thirty-two studies intervening at its source and five operating at the organisational or system level [32]. Future trials should therefore include outcomes that reflect both levels: symptom burden, guilt, shame and trust at the individual level; ethical climate, perceived institutional betrayal, moral residue, retention and team functioning at the organisational level.

Such studies also need careful timing. Moral injury may not be most visible during the acute event, when clinicians are occupied with action, but afterwards, when the event is appraised and incorporated into professional identity. Repeated lower-intensity episodes may be as important as single dramatic events. Longitudinal designs are therefore preferable to one-off surveys when the aim is to understand accumulation, recovery and the effect of organisational repair.

The second implication concerns measurement. Studies should declare the definition of moral injury used, justify the instrument selected and test whether its factor structure holds in the target population. Cross-cultural validation should be treated as a prerequisite rather than a technical appendix, particularly when instruments include religious or spiritual struggle. Role and specialty stratification should be built into the design from the outset. Samples labelled healthcare workers are no longer sufficient for a field that now claims clinical and policy relevance.

The third implication concerns psychiatry’s role. Psychiatry can help clarify symptom patterns, differential diagnosis and treatment needs, but it should not absorb moral injury into diagnostic categories without regard to causation. It can also contribute to occupational mental-health policy by distinguishing interventions that treat individual sequelae from those that repair workplace conditions. This distinction matters in any system in which resource allocation, professional hierarchy and institutional trust shape both clinical practice and clinician wellbeing.

A research agenda should therefore move in three directions: clearer construct definition, culturally calibrated measurement and intervention studies that treat organisational ethics as an outcome-relevant exposure. Moral injury is not only a problem of individual vulnerability; it is also a signal that professional conscience and institutional practice have diverged. That signal should be investigated before it is normalised.

### Limitations

The review has limitations. Restriction to the Web of Science Core Collection introduces an Anglophone and high-impact-journal bias. Co-citation analysis is also affected by citation lag: a reference must be cited, and cited together with others, before it can appear in the network at all. Only one burst begins in 2026 in these data, and the final slices are structurally incomplete rather than empty. This limitation bears directly on the specialty-gap thesis. Anaesthesiology and intensive care work on moral injury is recent, and a method that requires accumulated co-citation will under-detect it by construction; part of the gap reported in Section 3.6 is therefore an artefact of citation-detection lag rather than a genuine absence, and we are unable to quantify how much. What the data do support is the weaker but still consequential claim that, as of the search date, no specialty-specific work had become an organising reference for the field. Monographs and book chapters are underrepresented in document co-citation networks, despite their conceptual importance for moral injury. Finally, the clinical interpretation offered here is conceptual and should be tested in empirical studies stratified by profession, specialty, cultural setting and healthcare system.

## 5. Conclusions

In conclusion, moral injury identifies a form of clinician distress organised around moral transgression, betrayal and loss of trust rather than exhaustion alone. The moral injury literature has grown rapidly, but its definitions, instruments and clinical applications remain unevenly consolidated. Where clinicians are made to act against deeply held professional convictions by the conditions under which they work, the appropriate object of intervention is not only the clinician but the conditions. Specialty-specific work on moral injury exists in anaesthesiology and intensive care medicine, but as of the search date none of it had become an organising reference for the field, and part of that absence is attributable to citation lag rather than to a genuine gap in attention; connecting such work to the definitional and measurement debates that organise the literature is therefore a priority in its own right. The next phase of research should connect psychiatry, occupational mental health, ethics and health-services research to build definitions, measures and interventions adequate to that task.

## Figures and Tables

**Table 1:** Differential constructs related to clinician distress.

Dimension	Moral Injury	Moral Distress	Burnout	Compassion Fatigue/Secondary Traumatic Stress	PTSD
Origin/Key Authors	Shay (1994) [3]; Litz et al. (2009) [4]; Dean & Talbot (2019) [5]	Jameton (1984) [12]; Webster & Baylis (2000) [13]; Epstein & Hamric (2009) [14]	Maslach & Jackson (1981) [1]	Figley (1995) [15]	DSM-5-TR (2022) [17]
Definition	Lasting psychological harm from transgression of deeply held moral beliefs or betrayal by authority	Distress from knowing the right action but being constrained from taking it; cumulative moral residue across episodes	Syndrome of emotional exhaustion, depersonalisation, and reduced accomplishment	Emotional and physical exhaustion from sustained empathic engagement with suffering	Disorder following exposure to actual or threatened death, serious injury, or sexual violence
Core Mechanism	Violation of moral identity; betrayal of trust	Institutional or practical constraint on ethically correct action; accumulation of moral residue (crescendo effect)	Chronic imbalance between occupational demands and coping resources	Empathic overload through exposure to others’ suffering	Fear-based conditioning following threat exposure
Temporal Course	Often persistent and potentially cumulative; longitudinal evidence is limited	Situational; potentially reversible, but may accumulate (crescendo effect)	Gradual onset; chronic if conditions persist	Gradual or acute onset; potentially reversible with reduced exposure	Acute or delayed onset; variable course
Primary Focus	Proposed as systemic and situational	Situational (with systemic antecedents)	Individual–environment mismatch	Individual empathic capacity	Individual psychological response to threat
Principal Symptoms	Guilt, shame, self-condemnation, loss of trust, existential crisis	Anger, frustration, sense of powerlessness; moral residue	Emotional exhaustion, cynicism, inefficacy	Emotional exhaustion, reduced empathy, intrusive imagery	Re-experiencing, avoidance, hyperarousal, negative cognition and mood
Measurement Instruments	MISS-HP; MIES; MIOS	MDS-R	MBI	ProQOL; STSS	PCL-5; CAPS-5
Intervention Approach	Moral repair; systemic change; ethical infrastructure	Addressing institutional constraints; empowerment	Workload adjustment; resilience training; organisational change	Supervision; self-care; reduced exposure	Trauma-focused psychotherapy (CPT, PE, EMDR)

**Table 2:** Ten most co-cited references in the moral injury network (1961–2026).

Co-Citations	Reference	DOI	Cluster
269	Griffin BJ 2019 [7]	10.1002/jts.22362	#1
148	Čartolovni A 2021 [8]	10.1177/0969733020966776	#0
145	Mantri S 2020 [18]	10.1007/s10943-020-01065-w	#0
128	Litz BT 2022 [19]	10.3389/fpsyt.2022.923928	#0
128	Litz BT 2019 [21]	10.1002/jts.22405	#1
123	Hall NA 2022 [10]	10.1002/cpp.2607	#1
113	Bryan CJ 2018 [22]	10.1037/tra0000290	#1
110	Greenberg 2020 [20]	10.1136/bmj.m1211	#0
109	Williamson 2018 [9]	10.1192/bjp.2018.55	#0
96	Farnsworth JK 2017 [23]	10.1016/j.jcbs.2017.07.003	#1

**Table 3:** Summary of the ten largest clusters identified in the moral-injury co-citation network (1961–2026).

Cluster ID	Size	Silhouette	Label (LLR)	Label (LSI)	Mean Year
#0	245	0.707	moral distress	trauma-exposed black population	2021
#1	219	0.619	healthcare worker	religious strain	2019
#2	70	0.853	new challenge	psycho-spiritual development	2012
#3	62	0.752	combat-wounded veteran	suicide risk	2016
#5	49	0.917	military moral injury	mental health military	2015
#8	34	0.913	child protection service	social work research	2015
#9	31	0.980	cohort study	moderating role	2019
#16	13	0.991	conflict zone	civil protest movement	2023
#17	13	0.995	mechanism	moral injury: war-related trauma	2010
#52	4	0.998	mental health training	ineffective police leadership	2019

Abbreviations: LLR, log-likelihood ratio labelling; LSI, latent semantic indexing labelling. Mean year is the average publication year of the references within each cluster. Cluster identifiers are not consecutive because only the ten largest clusters are shown. Cluster labels are assigned by CiteSpace from the titles, abstracts and keywords of the articles citing a cluster and therefore characterise the citing community rather than the cited references constituting the cluster.

## Data Availability

The bibliographic records used for the scientometric co-citation analysis were retrieved from the Web of Science Core Collection (Clarivate), to which institutional access was available. The full reference list and the CiteSpace project file generated for this analysis are available from the corresponding author on reasonable request. No primary patient data were generated or analysed in this review.

## Supplementary Materials

The following supporting information can be downloaded at: https://www.mdpi.com/article/10.3390/healthcare14183075/s1, Supplementary Methods; Supplementary Table S1. Relevance classification of the 2765 source records, with the query as executed, the classification procedure and the full marker lists; Supplementary Table S2. Ten references with the strongest citation bursts (1961–2026); Supplementary Table S3. Presence of anaesthesiology, intensive care medicine, critical-care nursing, perioperative and pain medicine at three levels of the citation record. References [24,25,26,27,28,29,30,31,32,33,34,35,36,37] are cited in the supplementary materials.

## References

[1] Maslach C., Jackson S.E. (1981). The measurement of experienced burnout. J. Organ. Behav..

[2] Maslach C., Leiter M.P. (2016). Understanding the burnout experience: Recent research and its implications for psychiatry. World Psychiatry.

[3] Shay J. (1994). Achilles in Vietnam: Combat Trauma and the Undoing of Character.

[4] Litz B.T., Stein N., Delaney E., Lebowitz L., Nash W.P., Silva C., Maguen S. (2009). Moral injury and moral repair in war veterans: A preliminary model and intervention strategy. Clin. Psychol. Rev..

[5] Dean W., Talbot S., Dean A. (2019). Reframing clinician distress: Moral injury not burnout. Fed. Pract..

[6] Carey L.B., Hodgson T.J. (2018). Chaplaincy, spiritual care and moral injury: Considerations regarding screening and treatment. Front. Psychiatry.

[7] Griffin B.J., Purcell N., Burkman K., Litz B.T., Bryan C.J., Schmitz M., Villierme C., Walsh J., Maguen S. (2019). Moral injury: An integrative review. J. Trauma. Stress.

[8] Cartolovni A., Stolt M., Scott P.A., Suhonen R. (2021). Moral injury in healthcare professionals: A scoping review and discussion. Nurs. Ethics.

[9] Williamson V., Stevelink S.A.M., Greenberg N. (2018). Occupational moral injury and mental health: Systematic review and meta-analysis. Br. J. Psychiatry.

[10] Hall N.A., Everson A.T., Billingsley M.R., Miller M.B. (2022). Moral injury, mental health and behavioural health outcomes: A systematic review of the literature. Clin. Psychol. Psychother..

[11] Chen C. (2006). CiteSpace II: Detecting and visualizing emerging trends and transient patterns in scientific literature. J. Am. Soc. Inf. Sci. Technol..

[12] Jameton A. (1984). Nursing Practice: The Ethical Issues.

[13] Webster G.C., Baylis F., Rubin S.B., Zoloth L. (2000). Moral residue. Margin of Error: The Ethics of Mistakes in the Practice of Medicine.

[14] Epstein E.G., Hamric A.B. (2009). Moral distress, moral residue, and the crescendo effect. J. Clin. Ethics.

[15] Figley C.R. (1995). Compassion Fatigue: Coping with Secondary Traumatic Stress Disorder in Those Who Treat the Traumatized.

[16] Bride B.E., Robinson M.M., Yegidis B., Figley C.R. (2004). Development and validation of the Secondary Traumatic Stress Scale. Res. Soc. Work. Pract..

[17] American Psychiatric Association (2022). Diagnostic and Statistical Manual of Mental Disorders.

[18] Mantri S., Lawson J.M., Wang Z., Koenig H.G. (2020). Identifying moral injury in healthcare professionals: The Moral Injury Symptom Scale-HP. J. Relig. Health.

[19] Litz B.T., Plouffe R.A., Nazarov A., Murphy D., Phelps A., Coady A., Houle S.A., Dell L., Frankfurt S., Zerach G. (2022). Defining and assessing the syndrome of moral injury: Initial findings of the Moral Injury Outcome Scale Consortium. Front. Psychiatry.

[20] Greenberg N., Docherty M., Gnanapragasam S., Wessely S. (2020). Managing mental health challenges faced by healthcare workers during COVID-19 pandemic. BMJ.

[21] Litz B.T., Kerig P.K. (2019). Introduction to the special issue on moral injury: Conceptual challenges, methodological issues, and clinical applications. J. Trauma. Stress.

[22] Bryan C.J., Bryan A.O., Roberge E., Leifker F.R., Rozek D.C. (2018). Moral injury, posttraumatic stress disorder, and suicidal behavior among National Guard personnel. Psychol. Trauma..

[23] Farnsworth J.K., Drescher K.D., Evans W., Walser R.D. (2017). A functional approach to understanding and treating military-related moral injury. J. Context. Behav. Sci..

[24] Nash W.P., Marino Carper T.L., Mills M.A., Au T., Goldsmith A., Litz B.T. (2013). Psychometric evaluation of the Moral Injury Events Scale. Mil. Med..

[25] Shay J. (2014). Moral injury. Psychoanal. Psychol..

[26] Currier J.M., Holland J.M., Drescher K., Foy D. (2015). Initial psychometric evaluation of the Moral Injury Questionnaire-Military Version. Clin. Psychol. Psychother..

[27] Bryan C.J., Bryan A.O., Anestis M.D., Anestis J.C., Green B.A., Etienne N., Morrow C.E., Ray-Sannerud B. (2016). Measuring moral injury: Psychometric properties of the Moral Injury Events Scale in two military samples. Assessment.

[28] Drescher K.D., Foy D.W., Kelly C., Leshner A., Schutz K., Litz B. (2011). An exploration of the viability and usefulness of the construct of moral injury in war veterans. Traumatology.

[29] White S.M. (2021). Mental health, moral injury—And mandatory psychological assessment?. Anaesthesia.

[30] Hughes L., Shelley B., McPeake J. (2025). Exploring the experience of cardiothoracic ICU clinicians during the COVID-19 pandemic: A grounded theory study. Nurs. Crit. Care.

[31] Maben J., Taylor C., Dawson J., Leamy M., McCarthy I., Reynolds E., Ross S., Shuldham C., Bennett L., Foot C. (2018). A realist informed mixed-methods evaluation of Schwartz Center Rounds in England. Health Serv. Deliv. Res..

[32] Lang-Illievich K., Danninger T., Hammer S., Lang J., Szilagyi I.S., Bornemann-Cimenti H. (2026). Interventions to address moral distress in nurses and other healthcare professionals: A scoping review and evidence gap map. Healthcare.

[33] Currier J.M., Holland J.M., Malott J. (2015). Moral injury, meaning making, and mental health in returning veterans. J. Clin. Psychol..

[34] Frankfurt S., Frazier P. (2016). A review of research on moral injury in combat veterans. Mil. Psychol..

[35] Vargas A.F., Hanson T., Kraus D., Drescher K., Foy D. (2013). Moral injury themes in combat veterans’ narrative responses from the National Vietnam Veterans’ Readjustment Study. Traumatology.

[36] Gray M.J., Schorr Y., Nash W., Lebowitz L., Amidon A., Lansing A., Maglione M., Lang A.J., Litz B.T. (2012). Adaptive disclosure: An open trial of a novel exposure-based intervention for service members with combat-related psychological stress injuries. Behav. Ther..

[37] Farnsworth J.K., Drescher K.D., Nieuwsma J.A., Walser R.B., Currier J.M. (2014). The role of moral emotions in military trauma: Implications for the study and treatment of moral injury. Rev. Gen. Psychol..

