# Supplementary material for: Moral Injury Beyond Burnout: A Scientometric Review of Clinician Distress and Its Blind Spots in Critical Care
Source: Healthcare (Basel). 2026 Sep 18;14(18):3075. doi: 10.3390/healthcare14183075 (PMC13606641; doi:10.3390/healthcare14183075)
Supplement: Supplementary file 1 [file healthcare-14-03075-s001.zip › healthcare-4502093-supplementary.pdf]

Supplementary Information

Moral Injury Beyond Burnout: A Scientometric Review of Clinician Distress and Its Blind Spots in Critical Care

Helmar Bornemann-Cimenti, Istvan S. Szilagyi, Christoph Klivinyi, Kordula Lang-Illievich, Sascha Hammer

Manuscript healthcare-4502093

Contents

Supplementary Methods
Supplementary Table S1. Relevance validation of the 2,765 source publications
Supplementary Table S2. Ten references with the strongest citation bursts (1961–2026)
Supplementary Table S3. Presence of the specialty group at three levels of the citation record

Supplementary Methods

Network parameters and indicators

The g-index (k = 25) was selected as the node selection criterion, being the parameter most commonly applied in CiteSpace analyses of comparable scope. The link retaining factor (LRF = 2.5) and the look-back window (LBY = 5 years) filtered out single-instance co-occurrences while preserving established intellectual context; no network-level pruning was applied. Network quality was reported as modularity Q, with values above 0.3 indicating meaningful and above 0.7 strong cluster structure, and as the weighted mean silhouette score, with values above 0.7 taken to indicate a well-defined cluster structure. Sigma was calculated as (betweenness centrality + 1)^burst strength, the standard CiteSpace formulation. The Kleinberg burst-detection sensitivity parameter γ was set to 0.3 (default 1.0) to capture emerging bursts; of the 809 detected bursts, the 300 strongest were exported for further analysis.

Cluster labelling

To characterise the content of the clusters, three complementary methods of automated label generation were combined. The log-likelihood ratio (LLR) identifies statistically over-represented terms within a cluster and provides precise, discriminatory labels; it was used as the primary method. Latent semantic indexing (LSI) captures the dominant semantic context of a cluster and generates more descriptive, context-rich labels. Mutual information (MI) identifies terms with a high degree of specificity to a cluster and supplements the labels with particularly distinct terms. All three methods derive their labels from the titles, abstracts and keywords of the articles citing a cluster rather than from the cited references that constitute it, so that a label characterises the citing community rather than cluster membership.

Healthcare classification of citation bursts

Each detected burst was classified according to its healthcare relevance using a rule-based scheme. The reference text was checked against a predefined keyword list (including nurse, clinician, hospital, healthcare, worker, COVID, pandemic, burnout, moral distress, ethics) and a predefined list of journal-abbreviation stems (NURS, MED, JAMA, BMJ, LANCET, PSYCHIATR). The classification was automated rather than performed by human raters, so no inter-rater agreement statistic applies.

Reference selection: a four-criterion scientometric scheme

To move beyond a narrative selection of literature, the references discussed in the conceptual sections of this review were chosen on the basis of their position within the scientometric network. A reference was eligible if it satisfied at least one of four criteria: a co-citation count in the top 25 of the network, capturing sustained scholarly attention; a burst strength in the top 25, capturing phases of pronounced scholarly engagement; a sigma value in the top 10, capturing structurally transformative works that bridge clusters; or foundational monographic status, established by aggregated bibliography tracking across the citing articles in the two largest clusters. Standard diagnostic instruments and reference texts for the differential constructs (DSM-5, MBI, MDS-R, ProQOL, STSS, PCL-5, CAPS-5; Maslach 1981, Figley 1995, Bride 2004) were retained as background references, since they fall outside the moral-injury network but are required for construct differentiation. For the specialty-specific subsections (anaesthesiology, intensive care, critical care nursing) the scheme yielded too few references for substantive discussion; the consequent reliance on hand-searching is itself reported as a finding in the main text.

Protocol registration

The protocol was registered at the Open Science Framework (https://doi.org/10.17605/OSF.IO/NBKX9).

Table S1. Relevance validation of the 2,765 source publications.

Category	Definition	n	%
Explicit construct – phrase present	'Moral injury' or 'moral injuries' as phrase in title, abstract, author keywords or Keywords Plus	1,909	69.04%
Adjacent terms	'moral' and 'injur*' within 3 words of one another in any topic field	68	2.46%
Context-supported	Terms non-adjacent but with moral injury context markers (moral distress, moral trauma, PTSD, veteran, chaplaincy, healthcare worker, etc.)	87	3.15%
Mixed signals	Terms non-adjacent with both MI-context and contamination markers present	23	0.83%
Neutral	Terms non-adjacent, with no clear markers in either direction	441	15.95%
Likely contamination	Terms not adjacent with contamination markers only (workers' compensation, personal injury, sports/brain injury, moral judgement in child development, tort law)	237	8.57%
**Total**		**2,765**	**100%**

Contribution to the co-citation network. Of the 237 likely-contamination and 441 neutral records combined, none contributed above-threshold cited references to the network's largest connected component (0 of 740 network nodes originate from workers'-compensation, tort-law, sports-medicine, brain-injury or child-development literature).

Web of Science query as executed. The Web of Science Core Collection was searched on 20 April 2026 (17:55–17:57 UTC) using the topic query TS=(moral AND injur*). This Boolean-with-truncation query retrieves records in which the topic fields (title, abstract, author keywords, Keywords Plus) contain both the token 'moral' and any token beginning with 'injur' (matching 'injury', 'injuries', 'injurious'), with the two tokens permitted to occur non-adjacently. The strict-phrase alternative TS=("moral injury" OR "moral injuries") was considered and rejected because the validation reported above shows that 87 additional records (3.15% of the corpus) use the two tokens non-adjacently but discuss the moral-injury construct explicitly and would have been excluded by a phrase-only query. The publication-year window was 1961–2026; no restrictions on language or document type were applied at export. The following four Web of Science Core Collection indexes were selected: SCI-EXPANDED, SSCI, A&HCI, ESCI. The query returned 2,765 unique source publications (verified against the Web of Science accession number, UT field; 0 duplicates across the three export batches). Records were exported as tab-separated full records with cited references and imported into CiteSpace v7.0.R0 without further exclusion.

Classification procedure. Each record was classified against the six categories above using a rule-based, deterministic procedure applied to the concatenation of the title (TI), abstract (AB), author keywords (DE) and Keywords Plus (ID) fields, converted to lowercase. Categories were assigned in the order in which they appear in the table (Explicit construct → Adjacent terms → Context-supported → Mixed signals → Neutral → Likely contamination); a record was placed in the first category whose rule it satisfied. Category 1 required a match of the regular expression \bmoral\s+injur\w* or \bmoral-injur\w* anywhere in the concatenated topic fields. Category 2 required both a token matching \bmoral\w* and a token matching \binjur\w* with a whitespace-separated word distance ≤ 3 within the same topic field. Categories 3, 4 and 6 are defined by the presence of moral-injury-context markers, contamination markers, or both; Category 5 records contain neither.

Moral-injury-context markers. `The following case-insensitive regular expressions were counted as moral-injury-context markers: \bmoral distress\b, \bmoral pain\b, \bmoral wound\b, \bmoral trauma\b, \bmoral suffering\b, \bmoral repair\b, \bmoral damage\b, \bmoral disengagement\b, \bmoral outrage\b, \bmoral emotion\b, \bmoral residue\b, \bmoral transgression\b, \bmoral violation\b, \bmoral conflict\b, \bmoral dilemma\b, \bethical injury\b, \bcombat trauma\b, \bwar veteran\b, \bcombat veteran\b, \bptsd\b, \bpost.traumatic stress\b, \bcompassion fatigue\b, \bsecondary trauma\b, \bburnout\b, \bhealthcare worker\b, \bfrontline clinician\b, \bchaplain\b, \bethical (?:decision|dilemma|climate)\b, \bshay\b, \blitz\b.`

Contamination markers. `The following case-insensitive regular expressions were counted as contamination markers: \bbrain injury\b, \bspinal (?:cord)?injury\b, \bsports injury\b, \btraumatic brain injury\b, \btbi\b, \bhead injury\b, \bknee injury\b, \bacl injur\b, \bmoral (?:judg|reason|development)\w*\b, \bmoral panic\b, \bmoral hazard\b, \bworkers.compensation\b, \bpersonal injury\b, \btort\b, \bmusculoskeletal\b.`

Verification. The classification is deterministic given the input records and the two marker lists above. The categorised records are traceable via their Web of Science accession numbers (UT field), which are preserved in the deduplicated export deposited

with the CiteSpace project file at OSF (https://doi.org/10.17605/OSF.IO/NBKX9). Re-running the classification on the same input reproduces the counts reported above exactly.

Table S2. Ten references with the strongest citation bursts in the moral-injury network (1961–2026).

Burst strength	Reference	DOI	Cluster
66.77	Nash WP et al, 2013 [24], Mil Med, 178, 646	10.7205/MILMED-D-13-00017	#2
56.08	Shay J, 2014 [25], Psychoanal Psychol, 31, 182	10.1037/a0036090	#2
54.39	Currier JM et al., 2015 [26], Clin Psychol Psychother, 22	10.1002/cpp.1866	#2
37.89	Bryan CJ et al., 2016, Assessment [27], 23, 557	10.1177/1073191115590855	#2
37.77	Drescher K et al., 2011 [28], Traumatology, 17, 8	10.1177/1534765610395615	#2
36.38	Currier JM et al., 2015 [33], J Clin Psychol, 71, 229	10.1002/jclp.22134	#8
36.07	Frankfurt S, Frazier P, 2016 [34], Mil Psychol, 28, 318	10.1037/mil0000132	#1
35.02	Vargas AF et al., 2013 [35], Traumatology, 19, 243	10.1177/1534765613476099	#2
33.19	Gray MJ et al., 2012 [36], Behav Ther, 43, 407	10.1016/j.beth.2011.09.001	#2
33.12	Farnsworth JK et al., 2014 [37], Rev Gen Psychol, 18, 249	10.1037/gpr0000018	#2

Sigma combines burst strength and betweenness centrality and identifies references that are both structurally pivotal and have undergone phases of intensified scholarly attention; sigma values were highest for Currier et al. 2015 (Clin Psychol Psychother), Drescher et al. 2011 and Nash et al. 2013.

Table S3. Presence of anaesthesiology, intensive care medicine, critical care nursing, perioperative and pain medicine (the 'specialty group') at three levels of the citation record.

Level	Definition	n / N	%
Source publications	Records published in specialty-group journals	64 / 2,765	2.31%
Central cited references	References in the network's largest connected component (740 nodes) that were published in specialty-group journals	7 / 740	0.95%
Structurally transformative works	References among the 300 strongest citation bursts (Kleinberg $\gamma = 0.3$) that were published in specialty-group journals	0 / 300	0.00%
Source publications (supplementary check)	*Records published in specialty-group journals or with ≥1 author affiliated to a specialty-group department*	*81 / 2,765*	*2.93%*

Specialty-group inclusion criteria: journal titles containing ANAESTH, ANESTH, INTENSIVE CARE, CRITICAL CARE, PERIOPERATIVE or PAIN; author affiliations containing 'Department of Anesthesiology / Anesthesiology', 'Intensive Care Medicine', 'Intensive Care Unit / ICU', 'Critical Care Medicine', 'Critical Care Nursing', 'Perioperative', 'Pain Clinic', 'Pain Medicine' or 'Pain Management'. Classification was rule-based and automated on the exported Web of Science records (search executed 20 April 2026). The primary comparison uses a single criterion at all three levels, publication in a specialty-group journal, because author affiliations are not systematically available in the cited-reference records exported by Web of Science. The final row reports the affiliation-inclusive count for source publications as a supplementary check; it is not comparable with the second and third rows and is not part of the gradient.
